# Supplementary material for: Discovery and application of insertion-deletion (INDEL) polymorphisms for QTL mapping of early life-history traits in Atlantic salmon
Source: BMC Genomics. 2010 Mar 8;11:156. doi: 10.1186/1471-2164-11-156 (PMC2838853; doi:10.1186/1471-2164-11-156)
Supplement: Additional file 2 — Information on developed 76 locus single-run INDEL panel in Atlantic salmon. Information on fluorescence labeling, primer concentrations, PCR pooling and links to alignments, INDEL motifs and GENESCAN (Burge and Karlin 1997) predictions of genes/exons are available in html format. [file 1471-2164-11-156-S2.ZIP › Additionalfile2/snpsummary20411.html]

```
Cluster 10939 Contig 1

prev  Summary    Contig List  next
```

Size of Consensus sequence = 837

Number of sequences = 2

Minimum redundancy = 1

Key

A gi|85052526|gb|DW580704.1|DW580704 EST\_ssal\_rgb2\_45123 rgb2 Salmo salar cDNA clone ssal\_rgb2\_573\_018\_fwd 3', mRNA sequence  
B gi|89856772|gb|DY712895.1|DY712895 EST\_ssal\_rgb2\_68634 ssalrgb2 mixed\_tissue Salmo salar cDNA Salmo salar cDNA clone ssal\_rgb2\_611\_165\_fwd 3', mRNA sequence

5 SNPs detected

A B  cosegregation weighted

134 A C   5/5 100.00
247 A -   5/5 100.00
248 A -   5/5 100.00
249 T -   5/5 100.00
530 G C   5/5 100.00
